# Prophages and satellite prophages are widespread in *Streptococcus* and may play a role in pneumococcal pathogenesis

**DOI:** 10.1038/s41467-019-12825-y

**Published:** 2019-10-24

**Authors:** Reza Rezaei Javan, Elisa Ramos-Sevillano, Asma Akter, Jeremy Brown, Angela B. Brueggemann

**Affiliations:** 1 Nuffield Department of Medicine, University of Oxford, Oxford, UK; 2 UCL Respiratory, Division of Medicine, University College London, London, UK; 3 Department of Medicine, Imperial College London, London, UK; 4 Nuffield Department of Population Health, University of Oxford, Oxford, UK

**Keywords:** Bacteriophages, Bacterial genetics, Pathogens, Phage biology

## Abstract

Prophages (viral genomes integrated within a host bacterial genome) can confer various phenotypic traits to their hosts, such as enhanced pathogenicity. Here we analyse >1300 genomes of 70 different *Streptococcus* species and identify nearly 800 prophages and satellite prophages (prophages that do not encode their own structural components but rely on the bacterial host and another helper prophage for survival). We show that prophages and satellite prophages are widely distributed among streptococci in a structured manner, and constitute two distinct entities with little effective genetic exchange between them. Cross-species transmission of prophages is not uncommon. Furthermore, a satellite prophage is associated with virulence in a mouse model of *Streptococcus pneumoniae* infection. Our findings highlight the potential importance of prophages in streptococcal biology and pathogenesis.

## Abstract (web summary)

Prophages are viral genomes integrated within bacterial genomes. Here, Rezaei Javan et al. identify nearly 800 prophages and satellite prophages in > 1300 *Streptococcus* genomes, and show that a satellite prophage is associated with virulence in a mouse model of pneumococcal infection.

## Introduction

The genus *Streptococcus* comprises a wide variety of pathogens responsible for causing significant morbidity and mortality worldwide^1^. Some of the most important species causing disease in humans include: *Streptococcus pneumoniae* (pneumococcus), a leading cause of pneumonia, bacteraemia, and meningitis^2^; *Streptococcus pyogenes* (group A streptococci), a major cause of pharyngitis, scarlet fever and necrotising fasciitis^3^; and *Streptococcus agalactiae* (group B streptococci), the most common cause of neonatal sepsis^4^. In addition, *Streptococcus suis* and *Streptococcus equi* rarely cause disease in humans but are important animal pathogens^1^.

Bacteriophages (phages) are intracellular parasites of bacteria. Lytic phages hijack the host bacterial machinery, produce new phages and destroy the infected bacterial cell, whereas lysogenic phages do not necessarily initiate replication immediately upon host entry and may integrate their genome within the bacterial genome to be activated at a later stage. An integrated phage is termed a prophage and those genes can be passed down to the bacterial daughter cells. As survival depends on their bacterial hosts, prophages often express genes that increase host cell fitness^5,6^. Prophages can exert a range of phenotypic effects on the host bacteria: encode toxins that increase virulence^5^, promote binding to human platelets^7^ or cells^8^, evade immune defences^9,10^, or protect from oxidative stress^11^. Prophage integration can also regulate bacterial populations by altering bacterial gene expression^12,13^.

Prophages and their hosts, like other predator and prey relationships, are embroiled in a complex evolutionary arms race whereby bacteria evolve various strategies to defend themselves and prophages co-evolve to overcome these barriers^14^. These coevolutionary dynamics are complicated by satellite prophages, which lack all the necessary genetic information to replicate on their own and are reliant on hijacking the machinery of another inducing ‘helper’ prophage to replicate. Satellite prophages might be thought of as ‘parasites of parasites’^15,16^.

Satellite prophages adversely interfere with helper prophage replication and thus promote bacterial survival^17–19^. Satellite prophages have been discovered through different circumstances and thus there are different terms used to describe this particular type of mobile genetic element in the literature, including *Staphylococcus aureus* pathogenicity islands, phage-related chromosomal islands and phage-inducible chromosomal islands, among others^17–23^.

Satellite prophages have been shown to be vectors for the spreading of toxin genes and other virulence factors, e.g., SaPI1, which possesses the gene responsible for causing toxic shock syndrome^24^. The prevalence, diversity, genetic stability and molecular epidemiology of satellite prophages in streptococcal species are largely unknown. A small number of satellite prophages have been identified in streptococcal species, although whether they are associated with virulence remains to be investigated^25^. Previous work has shown that prophage-related sequences are highly prevalent within pneumococcal^26–28^, *S. pyogenes*^29,30^ and *S. agalactiae* genomes^31^; however, genus-wide analyses of the genomic diversity and population structure of streptococcal prophages have not yet been reported.

Here we report the discovery of ~800 prophages among >1300 streptococcal genomes and provide detailed insights into prophage genomics and population structure. Using the pneumococcus as the model organism, we investigate the molecular epidemiology of satellite prophages within a large globally-distributed collection of pneumococci isolated over a 90-year period and demonstrate that a satellite prophage is associated with virulence in a murine infection model.

## Results

### Prophages are a significant component of streptococcal genomes

**Fig. 1:**
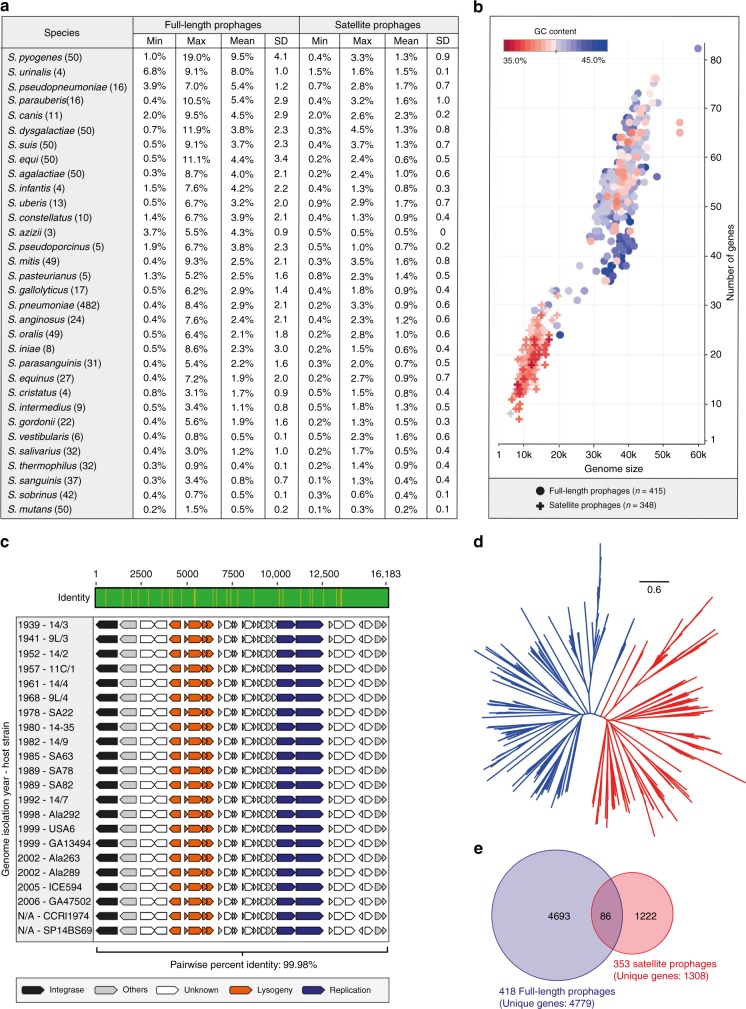
Prophages identified among streptococcal genomes. **a** Average prophage content within each streptococcal species. SD, standard deviation. **b** Graphical representation of all prophages by average genome size and number of genes. Each prophage is coloured to represent its average guanine (G) and cytosine (C) content. **c** Satellite prophage SpnSP24 was represented among pneumococci isolated between 1939 and 2006 and all of these satellite prophage sequences were nearly identical at the nucleotide level. **d** An unrooted phylogenetic tree of all streptococcal prophage genomes identified in the data set. Blue branches mark full-length prophages and red branches mark satellite prophages. **e** Venn diagram depicting the number of genes found exclusively in full-length prophages or in satellite prophages (at a threshold of >70% amino-acid sequence similarity) and those genes that are shared. Source data are provided as a Source Data file

We analysed 1306 genomes from 70 different streptococcal species and identified 415 full-length prophages and 348 satellite prophage genomes (Supplementary Data 1). We estimated the prophage gene content within each streptococcal genome and this revealed a substantial difference in the average prophage content among various streptococcal species, ranging from 0.4% of the *Streptococcus thermophilus* genome to 9.5% of the *S. pyogenes* genome (Fig. 1a; Supplementary Data 2). Furthermore, we observed significant variability in prophage content among different genomes of the same bacterial species, e.g., full-length prophages comprised up to 19% of the genes in some *S. pyogenes* genomes, whereas in others they made up <1% of the genome (Fig. 1a). The prevalence of satellite prophages ranged from 0.1% among *Streptococcus mutans* and *Streptococcus sanguinis* genomes to 4.5% of the *Streptococcus dysgalactiae* genomes (Fig. 1a).

### Full-length and satellite prophages are separate entities

Satellite prophages had a lower guanine (G) and cytosine (C) content than full-length prophages and were about a third of the size in terms of both length of sequence and the number of genes they harboured (Fig. 1b). Owing to their relatively small genome and apparent lack of essential genes, streptococcal satellite prophage sequences have often been regarded as “remnant” or “defective” prophages in a state of mutational decay^13,22,32–34^. Our data reveal that satellite prophage sequences can be highly conserved over many decades, e.g., one satellite prophage was present among pneumococcal genomes with isolation dates ranging from 1939 to 2006 and had maintained >99.98% nucleotide similarity across its entire genome (Fig. 1c), suggesting that it is under strong evolutionary pressure and likely provides an important biological function. The highly conserved nature of this satellite prophage is particularly striking given that the pneumococcus has long been known to be a highly recombinant organism^35,36^.

An unrooted phylogenetic tree of all streptococcal prophage genomes in our data set depicted full-length and satellite prophages as two clearly distinct groups (Fig. 1d). We observed that the genes of satellite prophages are unique and differ to those of full-length prophages, as 93% of all satellite prophage genes (>70% amino acid sequence similarity) are not found in any full-length prophages (Fig. 1e). Taken together, these findings confirm that satellite prophage sequences are not recent remnants of previous lysogenisation by full-length prophages, but rather that they belong to a unique family of mobile genetic elements.

### Streptococcal prophages have a structured population

**Fig. 2:**
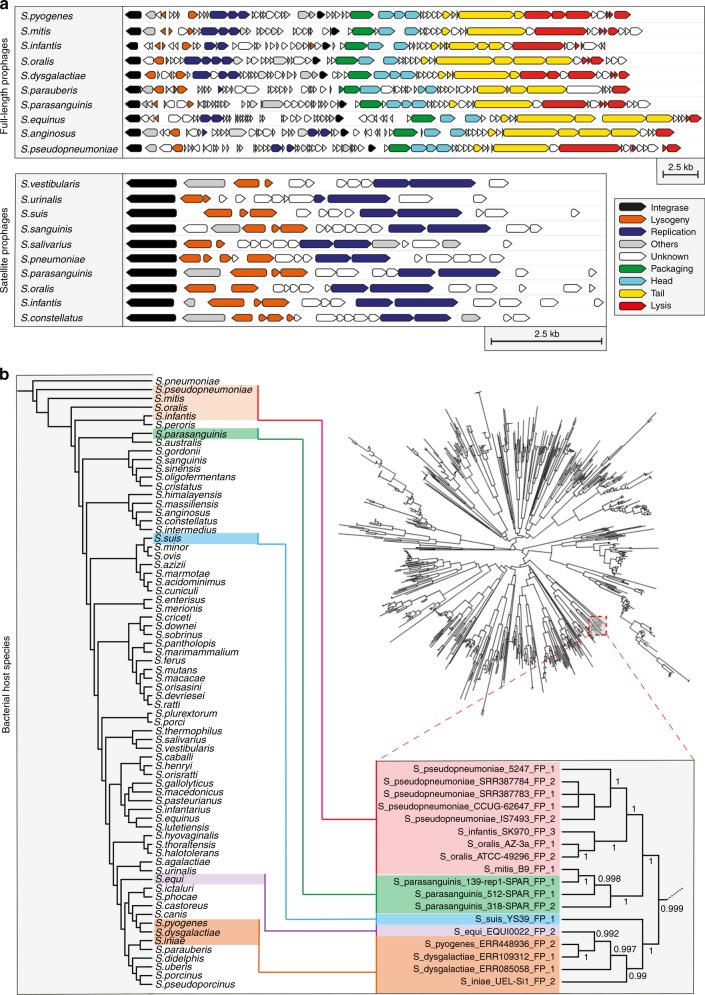
Evidence for cross-species transmission of prophages. **a** Full-length and satellite prophages identified among different streptococcal species shared a similar pattern in gene orientation and synteny. **b** Phylogenetic trees depicting the genetic relationships among streptococcal species (left) and all prophages detected in this study (right). A zoomed-in branch of the prophage tree (with branch lengths ignored for illustrative purposes) depicts one example of a cluster of full-length prophages that were found among multiple streptococcal species. Coloured boxes highlight where the indicated streptococcal species are found in each of the trees. A larger version of the tree is depicted in Supplementary Fig. 2 and a distance matrix of pairwise similarity among these 18 prophages is in Supplementary Fig. 3

We found that both full-length and satellite streptococcal prophages demonstrated well-conserved patterns in genome organisation and synteny, regardless of the species that they were isolated from (Fig. 2a). Similar to other non-streptococcal prophages (Supplementary Fig. 1), genes encoding specific functions were often found clustered together in the prophage genome, although note that the function of many genes is still unknown and therefore the delineation of discrete gene clusters remains problematic (Fig. 2a). Whole-genome comparisons of all prophage sequences in our data set depicted major and minor clusters for both full-length and satellite prophages (Fig. 2b; Supplementary Fig. 2).

Phages are generally believed to be bacterial species-specific and even specific to genetic lineages within a single bacterial species^37^. Surprisingly, we often found prophages from different bacterial species within the same phylogenetic cluster, suggesting that cross-species transmissions are more common among streptococcal prophages than previously realised. Remarkably, despite the relatedness of their prophages, the bacterial hosts were not necessarily the closest phylogenetically related species (Fig. 2b; Supplementary Fig. 3). One possible explanation could be that streptococcal prophages are evolving separately from their microbial hosts, and therefore, other factors such as ecological relatedness may dominate over evolutionary relatedness of the host bacteria.

### Molecular epidemiology of pneumococcal satellite prophages

We had previously determined the prevalence, diversity and molecular epidemiology of full-length prophages in a global and historical pneumococcal genome data set^26^. Many shorter prophage sequences were also identified in that study, which were simply classified as partial prophage sequences and not characterised further at the time. Here, we used this genome data set to further investigate satellite prophages in the context of the pneumococcal population structure. The genome collection was comprised of 482 pneumococci recovered from both healthy and diseased individuals between 1916 and 2009. Pneumococci were isolated from people of all ages residing in 36 different countries. Ninety-one serotypes and 94 different clonal complexes (genetic lineages) were represented in the data set.

**Fig. 3:**
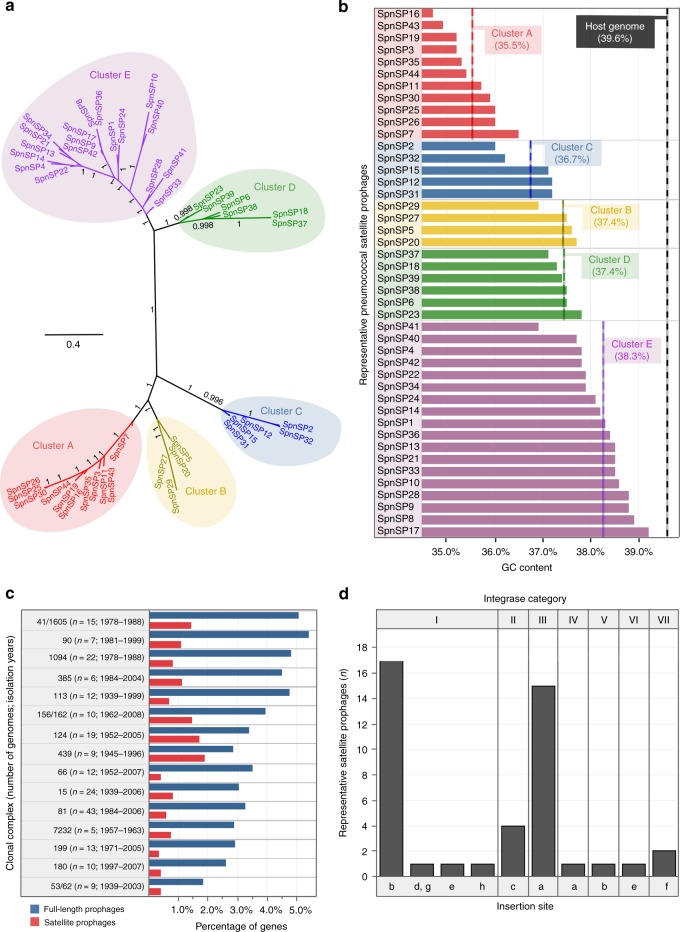
Satellite prophages among pneumococci. **a** An unrooted phylogenetic tree demonstrated that the 44 representative satellite prophages could be clustered into five major groups based upon nucleotide similarity. **b** The average guanine/cytosine (GC) content (stated in brackets) of the satellite prophages varied by genetic cluster and was lower than the GC content of the pneumococcal host. **c** The average prophage content for each of the major clonal complexes (genetic lineages) is depicted as a percentage of the total number of genes in the host pneumococcal genome (∼2 Mb). **d** The integrase sequences of the 44 representative satellite prophages were divided into seven different categories based upon ≥95% nucleotide similarity. Source data are provided as a Source Data file

A reinvestigation of the ‘partial prophage’ sequences resulted in the identification of 44 representative pneumococcal satellite prophages, which clustered into five major groups (Fig. 3a). The average GC content of the satellite prophages was lower than their pneumococcal host but varied among each group (Fig. 3b). We found that 35% of the pneumococci in our data set contained at least one satellite prophage and 5% of the genomes contained two. Some satellite prophages were present in up to six different clonal complexes, whereas others were only found in Singletons (genotypes with no closely related variants; Table 1 and Supplementary Fig. 4). Those satellite prophages identified in more than one genome were often found among pneumococci recovered over a decade or more (Table 1). The average prophage content for each of the major clonal complexes ranged from 2.2 to 6.5%, and with only one exception (CC7232), all of these are widely circulating pneumococcal genetic lineages (Fig. 1c; https://pubmlst.org/spneumoniae).

**Table 1:** Epidemiological characteristics of 44 satellite prophages identified among pneumococci

Prophage	Cluster	Isolation	Genomes (*n*)	CC (*n*)	Country (*n*)	Serotype (*n*)	Site	Int
SpnSP16	A	1939–1982	4	3	2	4	b	I
SpnSP3	A	1981–2004	3	2	2	2	b	I
SpnSP26	A	1985–2000	3	2	1	2	b	I
SpnSP35	A	1952–1952	2	2	1	2	b	I
SpnSP43	A	1939–2004	2	2	2	2	b	I
SpnSP30	A	1978–1978	5	1	1	1	b	I
SpnSP44	A	1939–1962	2	1	1	1	b	I
SpnSP7	A	1968	1	1	1	1	b	I
SpnSP25	A	1999	1	1	1	1	b	I
SpnSP19	A	1939–1952	2	S	2	2	b	V
SpnSP11	A	1952	1	S	1	1	b	I
SpnSP5	B	1939–2007	15	5	3	7	d, g	I
SpnSP29	B	1978–1988	15	1	1	2	b	I
SpnSP27	B	2006	1	1	1	1	b	I
SpnSP20	B	1954	1	S	1	1	b	I
SpnSP2	C	1984–2005	4	2	3	2	f	VII
SpnSP31	C	1983–2005	2	2	1	2	b	I
SpnSP12	C	1968	1	1	1	1	b	I
SpnSP15	C	1943	1	1	1	1	b	I
SpnSP32	C	1986	1	1	1	1	f	VII
SpnSP37	D	1939–1988	9	5	4	7	c	II
SpnSP38	D	1972–2006	30	4	6	5	c	II
SpnSP6	D	1939–1991	8	3	3	3	c	II
SpnSP23	D	1962–2008	11	2	3	4	a	III
SpnSP39	D	2005–2007	2	1	1	1	a	III
SpnSP18	D	1939–1952	2	S	2	2	c	II
SpnSP24	E	1939–2006	23	6	6	4	a	III
SpnSP33	E	1952–1998	3	2	1	2	a	III
SpnSP1	E	1978–1988	5	1	1	1	b	I
SpnSP40	E	2001	3	1	2	2	a	III
SpnSP8	E	1988	1	1	1	1	a	III
SpnSP9	E	1957	1	1	1	1	a	III
SpnSP13	E	1943	1	1	1	1	a	III
SpnSP14	E	1995	1	1	1	1	a	III
SpnSP17	E	1972	1	1	1	1	a	IV
SpnSP22	E	1971	1	1	1	1	a	III
SpnSP28	E	2003	1	1	1	1	a	III
SpnSP34	E	1990	1	1	1	1	a	III
SpnSP36	E	1963	1	1	1	1	a	III
SpnSP42	E	1994	1	1	1	1	a	III
SpnSP4	E	1982	1	S	1	1	e	I
SpnSP10	E	N/A	1	S	1	1	h	I
SpnSP21	E	1954	1	S	1	1	e	VI
SpnSP41	E	1983	1	S	1	1	a	III

Note: Prophage = name of each satellite prophage; Cluster = satellite prophage cluster (see Fig. 3); Isolation = isolation date(s) of the pneumococci that harboured the satellite prophage; Genomes = number of pneumococcal genomes in which the satellite prophage was identified; CC = clonal complex (genetic lineage) of the host pneumococcus; Country = the number of countries in which the pneumococci were recovered; Serotype = number of different serotypes of the host pneumococci; Site = prophage insertion site within the pneumococcal genome (see Fig. 4); Int = integrase sequence of the satellite prophage; S = singleton, a genotype with no closely related variants

### Prophages and satellite prophages have defined integration sites

**Fig. 4:**
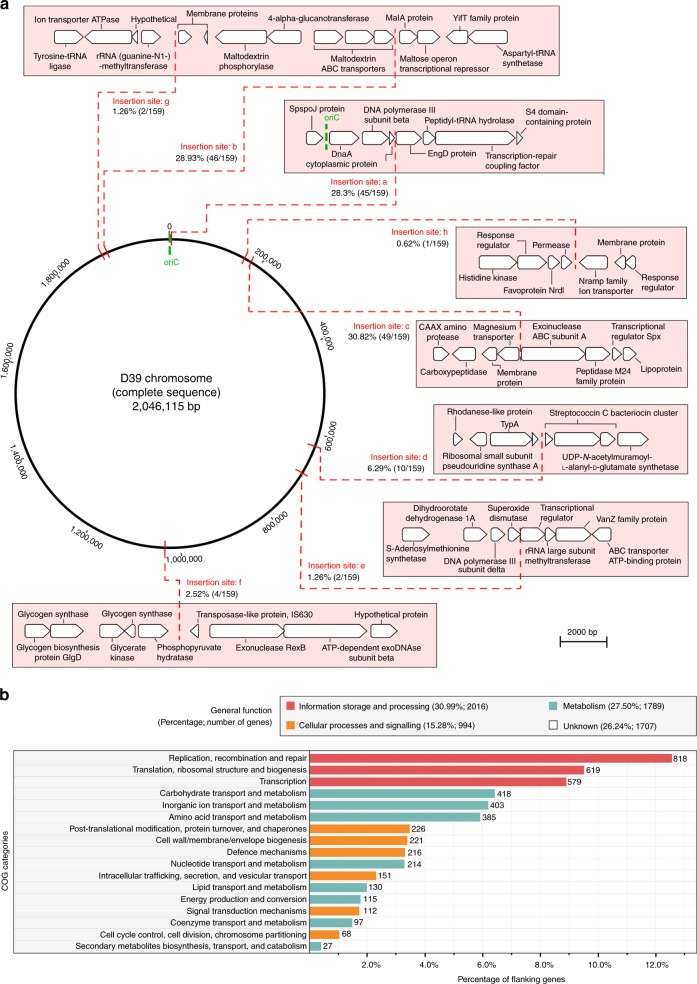
Insertion sites of prophages. **a** Pneumococcal satellite prophages were integrated in seven locations (**a**–**f**) within the host genome. Percentages and numbers in brackets refer to the proportion and number out of all 159 satellite prophages that were inserted in that particular location. **b** The flanking genes upstream and downstream of all integrated full-length and satellite prophages within the streptococcal genomes were retrieved for functional classification and are depicted here based upon their COG (clusters of orthologous groups) classifications

We previously reported that pneumococcal full-length prophages were consistently integrated in specific locations within the genome^26^. Likewise, pneumococcal satellite prophages were consistently integrated in seven precise locations (a–f) within the host genome, each of which was directly associated with the integrase gene they harboured (Fig. 3d; Fig. 4a). The 44 representative satellite prophage integrases were divided into seven different categories with ≥95% nucleotide sequence similarity within each category. Each integrase category was associated with insertion at a single location on the pneumococcal genome, apart from integrase category I, which was associated with five different locations (Fig. 3d). In all, 28.3% of pneumococcal satellite prophages were inserted at site a, which was very close to the origin of replication (oriC) (Fig. 4a) and prompted us to investigate whether factors other than the integrase sequence determined the prophage insertion site.

We investigated the location of prophage insertion sites within the genome sequences of non-pneumococcal streptococci for which at least one complete (finished) genome was available (*n* = 29). We divided the genome of each species into eight non-overlapping segments of equal length according to the number of base pairs, and the percentages of prophages situated in each segment were quantified. Overall, we observed no strong preference for prophage insertion in any of the eight segments and the location of prophages residing within the genome varied greatly between different species (Supplementary Fig. 5).

Among pneumococcal and non-pneumococcal streptococcal genomes, five flanking genes upstream and downstream of each prophage were retrieved for functional classification using gene ontology analyses. This revealed that nearly one-third of all the bacterial flanking genes were involved in replication, recombination, DNA repair, transcription, translation and ribosomal structure and biogenesis (Fig. 4b). One-quarter of flanking genes were involved in metabolic processes, but equally, one-quarter of all flanking genes did not have a defined functional classification. The remaining flanking genes were involved in other cellular processes and signalling. A list of all prophage insertion sites and their flanking genes is available in Supplementary Data 3.

For comparison, we selected one genome of each of the 70 different streptococcal species, determined the clusters of orthologous groups (COGs) for all streptococcal genes, and then compared those genome-wide streptococcal data to the COGs represented by the prophage flanking genes in the overall data set. This demonstrated that the distributions of COGs categories were significantly different, and while prophage flanking genes were more likely to be in the information storage and processing COGs category, the most prevalent COGs category among all streptococcal genes was metabolism (32.1% of all genes; Supplementary Table 1).

### Satellite prophages and *vapE* are involved in pathogenesis

**Fig. 5:**
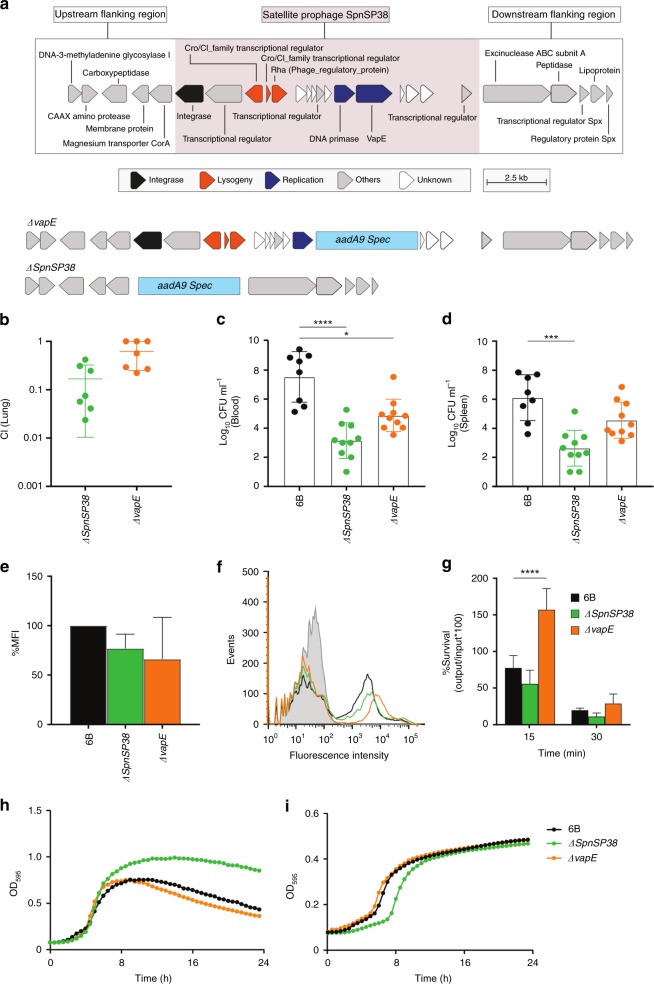
A satellite prophage is associated with virulence. **a** Upper part depicts the satellite prophage genes integrated within the BHN418 genome and flanking pneumococcal genes, and the lower part depicts the *∆vapE* and *∆SpnSP38* mutants with the addition of the spectinomycin resistance cassette aadA9. **b** Plots of the competitive index (CI) for the *∆SpnSP38* and *ΔvapE* mutant strains versus the wild‐type strain in a mouse model of pneumonia. Each symbol represents the CI for a single animal and bars represent the median and range. **c**, **d** Mean bacterial colony-forming units (CFU) recovered at 24 h from blood (**c**) or spleen (**d**) homogenates after intraperitoneal inoculation of 5 × 10^6^ CFU/strain. Each symbol represents data for a single animal. **e** Mean fluorescence intensity (MFI) of C3b deposition on the surface of the wild-type and mutant strains as measured by a flow cytometry assay. **f** Example of a flow cytometry histogram for the C3b deposition data. **g** Bacterial survival in a neutrophil-killing assay (multiplicity of infection: 1 bacterium/100 neutrophils) represented as % CFU/ml recovered after 15–30 min incubation compared with the input bacteria. **h**, **i** Growth curves as measured by the optical density (OD) of wild-type and mutant strains cultured in Todd-Hewitt broth supplemented with 0.5% yeast-extract (**h**) or 100% human serum (**i**). Error bars **c**, **d**, **e**, **g** represent standard deviation and asterisks **c**, **d**, **g** represent statistical significance compared with the wild-type strain (two-sided Kruskal–Wallis test with Dunn’s correction for multiple comparisons) *p < 0.05; ***p < 0.001; ****p < 0.0001). Source data are provided as a Source Data file

Our investigation of pneumococcal satellite prophage genes led to the identification of a gene that is a homologue of the ‘virulence-associated gene E’ (*vapE*) in *S. suis*^38^. We investigated *vapE* in *S. suis* genomes and confirmed that it is carried by a satellite prophage. We searched for *vapE* in the representative pneumococcal satellite prophages and found that 30/44 (68.2%) contained *vapE*. To investigate whether the *vapE* homologue in the pneumococcal satellite prophage is also associated with virulence, we performed in vivo studies using a murine pneumococcal infection model and one example of a satellite prophage containing *vapE* identified in this study (Fig. 5a).

Deletion mutant stains were constructed in a serotype 6B pneumococcal strain, BHN418, which contains a satellite prophage sequence (SpnSP38; GenBank accession number MK448645) and no full-length prophage sequences (see Supplementary Data 4 for details of the gene content of BHN418). Either *vapE* only (*∆vapE*) or the entire satellite prophage sequence (*∆SpnSP38)* were replaced by a spectinomycin resistance cassette (*aadA9*) in the BHN418 strain (Fig. 5a). For each of the mutant strains a competitive index (CI) was determined using a highly sensitive competitive infection experiment in a mouse model of pneumonia.

The CI was significantly <1 in the lungs after mixed infection with *∆SpnSP38* and the wild-type serotype 6B or *∆vapE* and the wild-type serotype 6B, indicating a role for the satellite prophage and *vapE* in the establishment of pneumococcal pneumonia (Fig. 5b). To further assess the degree of attenuation in virulence of the *∆SpnSP38* and *∆vapE* strains, infection experiments were repeated with pure inocula of each strain in both the pneumonia and sepsis models. There were no significant differences in bacterial CFU recovered from the lungs of infected mice at 24 h between either mutant and the parental wild-type strain and the majority of the mice developed fatal infection by this point. However, in the sepsis model, the mice infected with the wild-type serotype 6B strain had significantly greater blood and spleen CFU than the *∆SpnSP38* mutant (Fig. 5c, d), indicating that the satellite prophage is directly involved in pneumococcal virulence during bacterial dissemination in the systemic circulation. Although the *∆vapE* strain had lower spleen CFU compared with the wild-type, this difference was not statistically significant, suggesting that loss of the whole-satellite prophage has a more marked effect on the attenuation of virulence during sepsis than loss of VapE alone.

### The satellite prophage is required for optimum growth in sera

Reduced systemic virulence of *∆SpnSP38* or *∆vapE* mutants could reflect poor growth under physiological conditions, or evasion of host innate immune killing, which is largely dependent on complement-mediated neutrophil killing. Using a flow cytometry assay, the binding of complement component C3b was not demonstrably different between the mutant strains and wild-type strain (Fig. 5e, f). Furthermore, survival of the *∆SpnSP38* and *∆vapE* mutants in the presence of neutrophils after 30 min was similar to the wild-type BHN418 strain (Fig. 5g). These data indicate that the satellite prophage and *vapE* are not required for evasion of complement or neutrophil killing, and that the reduced virulence of the *∆SpnSP38* strain could reflect delayed growth in serum. Growth rates of both mutant strains in THY were not significantly different to the parental wild-type strain (Fig. 5h); however, culture in serum demonstrated a small but significant delay in growth of the *∆SpnSP38* strain compared with the wild-type and ∆*vapE* strains (Fig. 5i).

### Satellite prophage genes were overexpressed in planktonic culture

Given the association of the satellite prophage and *vapE* with virulence in our murine pneumococcal infection model, we hypothesised that satellite prophage genes would be overexpressed when pneumococci were grown planktonically in broth versus in a biofilm. To evaluate this hypothesis, we performed comparative transcriptome analyses of planktonic and biofilm pneumococci using an existing RNA sequencing data set generated by Blanchette et al.^39^. In their study, pneumococcal reference strain Sp6A-10, which contained two full-length prophages and one satellite prophage (SpnSP33, 58.7% identical to SpnSP38; GenBank accession number MK448640), was grown planktonically and as a 2-day-old biofilm. Three biological replicates were collected from each of the growth conditions and the corresponding RNA samples were extracted and sequenced.

**Fig. 6:**
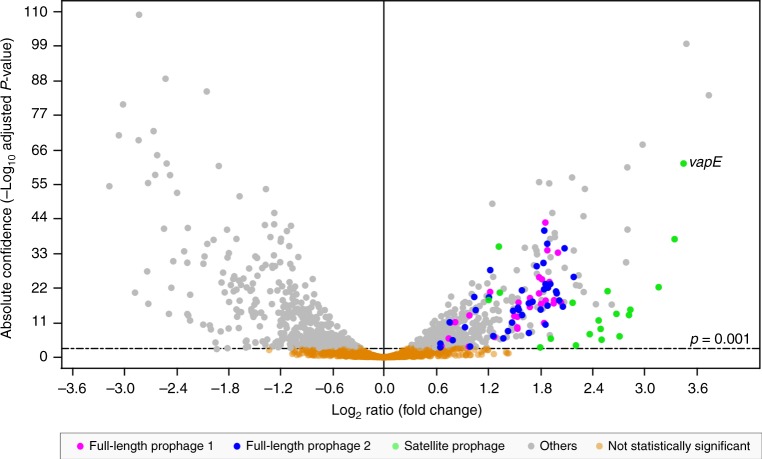
Differential expression of satellite prophage genes. The data were generated from pneumococcal reference strain Sp6A-10, which contains two full-length prophages (Spn_6A-10_FP1 and Spn_6A-10_FP2) and one satellite prophage (SpnSP33). Genes belonging to SpnSP33 are shown in green, while those belonging to Spn_6A-10_FP1 and Spn_6A-10_FP2 are shown in blue and magenta, respectively. Differential gene expression and statistical significance were computed using the DESeq2 method^70^. The dashed line above the *x* axis marks the significance threshold of *p* = 0.001. A higher log_2_ ratio denotes increased expression levels in planktonic growth as compared with growth in a biofilm. A full list of the genes depicted here, their expression levels and sequences may be found in Supplementary Data 5. The annotated genes and relative expression levels of all three prophages are found in Supplementary Fig. 6

We analysed the Blanchette transcriptomic data^39^ to assess prophage gene expression under these two experimental conditions, and the data demonstrated significantly higher satellite prophage and full-length prophage gene expression when the host pneumococcus was grown in broth as compared with growth in a biofilm (Fig. 6; Supplementary Data 5). The full complement of satellite prophage genes were significantly expressed, and many of the genes of the two full-length prophages, mainly structural and lysis genes, were also significantly upregulated. These gene expression patterns were consistent with the hypothesis that the satellite prophage was exploiting the other full-length prophages in the pneumococcal genome as helper prophages, since the satellite prophage does not possess phage structural genes.

Notably, among the 20 most significantly upregulated genes, 60% (*n* = 12) were satellite prophage genes and *vapE* was the third most upregulated gene in the entire genome. Among the 50 most highly expressed genes, just over half were prophage-related genes: 15 (30%) were satellite prophage genes; 7 (14%) were genes of one full-length prophage; and 4 (8%) were genes of the second full-length prophage (Fig. 6; Supplementary Fig. 6; Supplementary Data 5). These experimental data further support a significant role for satellite prophages and *vapE* (and full-length prophages) in pneumococcal biology.

## Discussion

In this study, we sampled a large collection of streptococcal genomes and revealed a diverse collection of full-length prophages and satellite prophages among streptococcal species. What was striking about these findings was that prophages and satellite prophages were two clearly different entities and both had a structured population. Specifically, among pneumococci there were full-length prophages and satellite prophages with persistent associations to major, epidemiologically successful genetic lineages of pneumococci over long periods of time. This is crucial, as these data allow for the exploration of why certain combinations of prophages and bacteria exist and whether the prophages might be contributing to the epidemiological success of bacterial genetic lineages.

Our findings suggest that prophages are likely to be influencing bacterial biology and epidemiology to a much greater extent than previously appreciated, given the high proportion of prophage DNA present in many streptococcal species—many of which have not previously been analysed for evidence of prophages. Prophages are mobile genetic elements and genetically similar prophages were frequently detected between different streptococcal species. Historically, the prevailing dogma is that phages have a narrow host range, but our data challenge this view and suggest that prophage transmission across bacterial species is more common than previously recognised. Other investigators have also recently suggested that some phages may have a broader host range than previously appreciated^40^.

Our data set was designed to be comprised of streptococci that were genetically different and geographically widely distributed, rather than from a very defined population. These data demonstrated high prophage diversity overall, given the breadth and depth of the data set, and what was remarkable was the similarity among prophages in different bacterial species. In the context of a highly diverse data set, there are two plausible explanations for finding the same or highly similar prophages in different species, the most likely of which is cross-species transmissions of prophages, or at least prophage sequences. The alternative explanation is a shared common ancestor, but this is far less likely given the overall variation among prophage sequences, at least on any reasonable time frame. The implications of these findings are that host specificity should be taken into account when trying to understand the precise role of prophages in streptococcal biology and when considering whether phages might be used in any therapeutic interventions.

Many of the streptococci we investigated are important human and animal pathogens, raising the question whether prophages influence host virulence potential. To investigate this, we assessed the effects of deleting a pneumococcal satellite prophage sequence on virulence in a murine model of infection. This prophage contains *vapE*, a gene that has previously been described to have a role in *S. suis* virulence through an unknown mechanism. The results showed that deletion of the whole prophage or *vapE* alone had a significant effect on pneumococcal virulence, and deletion of the whole prophage had a particularly strong effect and reduced recovered CFU for the sepsis model by approaching 10^4^ log_10_. In vitro characterisation of the mutant strains indicated that the reduced virulence of the prophage mutant was related to impaired growth in serum rather than avoidance of opsonophagocytic killing. How the prophage influences pneumococcal growth in serum will require more detailed investigation, but the stronger phenotypic effect of loss of the whole prophage compared with *vapE* alone suggests that additional prophage genes are involved in virulence. For example, the prophage is predicted to contain regulatory genes, which could potentially improve growth in serum by altering the expression of metabolic and transporter genes.

Furthermore, when we analysed the transcriptomic data from Blanchette et al.^39^, these data demonstrated that all satellite prophage genes (including *vapE*) and many genes of the two full-length prophages were among the most significantly upregulated among pneumococci growing in planktonic form (which is akin to pneumococcal bacteraemia) rather than in a biofilm (a state in which pneumococci are less likely to be virulent^41^). Although the specific mechanism driving virulence is not yet clear, this work provides clear evidence that experimental investigations of pneumococcal prophages and satellite prophages can reveal central aspects of the bacteria/prophage relationship among pneumococci and other streptococci.

The increasingly large volume of genome sequence data in the public domain presents many opportunities for understanding bacterial infection and pathogenesis at a depth and breadth never before experienced. Large population-level analyses such as this alter our perspective on how bacterial and prophage populations interact and drive evolution of both parasite and host. As demonstrated here, population genomics studies can and should be used to generate hypotheses, design experiments, and select the most appropriate strains for testing. The findings of this study reveal numerous areas for further investigation, the results of which will increase our knowledge of prophage and bacterial biology, epidemiology and evolution.

## Methods

### Development of PhageMiner for prophage identification

Some in silico prophage detection tools are available that identify prophages by comparison with a reference database of known prophage genomes, thus their performance is strongly influenced by the size and composition of the reference data set^42,43^. In order to ensure a thorough discovery of previously unidentified prophages, manual curation of annotated genomes is required, however, this is not feasible for large genome studies^26,44,45^. To address these issues, we developed a user-supervised semi-automated computational tool called PhageMiner in order to streamline the manual curation process for prophage sequence discovery.

The PhageMiner pipeline consists of a series of steps, as follows. The bacterial genome of interest is annotated using the RAST annotation server (http://rast.nmpdr.org) in order to create an annotated GenBank file, which is then input into the PhageMiner Python script. The location and the annotated name of each ORF in the host genome is retrieved from the annotated GenBank file and saved to a comma-separated value (CSV) file using the Biopython package (http://biopython.org). A number of predefined user-adjustable phage-associated keywords are then used to scan the CSV file generated in the previous step and any ORF containing a matching string (e.g., “phage”, “lytic amidase”, “tail fibre protein”, etc.) in its annotation name is deemed a ‘hit’. An additional set of predefined user-adjustable keywords are used to discard any matching hits with annotation names that resemble phages but are not prophage genes (e.g., ‘macrophage’). Using the mean shift clustering method in Scikit-Learn machine learning library (https://scikit-learn.org), the location of the remaining phage hits respective to each other and to the size of the host genome are used to identify clusters of bacteriophage-related genes. During this step, minimal manual inputs by the user are requested in order to ensure correct identification of prophage regions. If necessary, clustering can be repeated with a different sensitivity as redefined by the user, or alternatively, the coordinates corresponding to each suspected prophage region can be entered manually. The pipeline is aborted at this stage if no clusters of bacteriophage-related genes are detected or manually defined by the user. Once clusters of bacteriophage-related genes are identified, PhageMiner creates various figures and tables related to each of the suspected prophage regions, the most important of which are a schematic diagram of the coding regions, the location of the prophage region in the chromosome including the flanking genes adjacent to the prophage region, the presence of any assembly gaps, and the nucleotide sequences of the ORFs in the cluster. If necessary, the number of flanking genes displayed in each figure can be manually adjusted. Based on the decisions made by the user, the putative prophage genomes are either rejected or extracted as a separate GenBank file and categorised into three groups: full-length prophages, satellite prophages and unknown phage-related regions. The source code of PhageMiner is available from GitHub.

### Genomes used in this study

In total, 1306 assembled genomes from 70 different species of the genus *Streptococcus* were selected for this study, of which 482 genomes belonged to a pneumococcal data set previously characterised by us^26^. This collection was designed to be highly diverse and consisted of pneumococci recovered from both ill and healthy individuals of all ages residing in 36 different countries between 1916 and 2009. These pneumococci represented 91 serotypes and 94 different clonal complexes (Supplementary Data 6).

The remaining 824 streptococcal genomes were selected from a non-pneumococcal *Streptococcus* species genome data set previously compiled by us^46^. In brief, 69 different *Streptococcus* species were included in this data set and up to 50 genomes per species were selected for analyses from the ribosomal MLST database (https://pubmlst.org/rmlst)^47^. When >50 genomes were available, the population structure of the species was depicted using PHYLOViZ^48^ and genomes were selected to maximise the population-level diversity of the species from the available genomes. All streptococcal genome sequences were stored in a BIGSdb database^49^ and annotated using the RAST server (http://rast.nmpdr.org).

### Sequence analyses of prophages

All putative prophage sequences were inspected manually using Geneious version 11.1 (Biomatters Ltd; https://www.geneious.com) and those containing ambiguous bases (N’s) and/or assembly gaps (*n* = 411) were excluded from further analyses. The total number of open reading frames (ORFs), overall sequence length and GC content of each prophage were calculated within the Geneious environment. All multiple sequence alignments were performed using ClustalW (version 2.1)^50^ with default parameters (Gap open cost = 15, Gap extend cost = 6.66). Phylogenetic trees were constructed based upon sequence alignments using FastTreeMP (version 2.1.5)^51^. Unique integrase sequences were identified using the CD-HIT programme (version 4.6.6)^52^ and a threshold of ≥95% sequence identity. Schematic diagrams of the coding regions of the prophages were produced in Geneious and edited using Adobe Illustrator.

### Estimation of prophage content within bacterial genomes

The phage content was estimated based on the percentage of prophage genes within a given bacterial genome. To do this, we developed a Python script that first used Prodigal software in the Prokka annotation suite (version 1.10)^53^ to predict ORFs in three separate groups of sequences: (i) all identified full-length prophage genomes, (ii) all identified satellite prophage genomes and (iii) a single bacterial genome of interest for which the phage content is to be estimated. Next, the individual ORF nucleotide sequences from all three groups were extracted, combined and clustered using Roary^54^ set at a 70% similarity threshold. Any ORFs in the bacterial genome that were also present in at least one prophage genome were deemed to be phage-related, and this information was used to output the total percentage of phage-related ORFs in the given bacterial genome. The PhageContentCalculator script is available from GitHub.

### Investigation of prophage insertion sites and flanking genes

The prophage insertion sites within the bacterial genomes were investigated among the representative pneumococcal prophages and any streptococcal species for which at least one complete bacterial genome was available. Prophage insertion sites containing ambiguous bases or assembly gaps were excluded from the analyses. In order to assess the relative location of prophages within streptococcal bacterial genomes, the genomes were divided into eight equally sized segments and the prevalence of prophages per segment was calculated.

To investigate the location of prophages relative to the putative function of the flanking bacterial genes, the sequences of the five bacterial genes both upstream and downstream of each prophage were retrieved. Bacterial gene sequences were categorised into COGs using eggNOG-mapper, which is based on eggNOG 4.5 orthology data^55,56^. For comparison, a reference set of 70 streptococcal genomes, each representing a different streptococcal species, was compiled. All bacterial genes were assigned a COGs category using eggNOG and the average prevalence of each COG category across the combined set of 70 reference streptococcal genomes was calculated.

### Construction of a pneumococcal core genome phylogenetic tree

The 482 pneumococcal genomes in the study data set were annotated using Prokka in order to create GFF3 files compatible with downstream analysis scripts. Genes present in all strains were clustered at 90% sequence identity threshold and aligned using Roary. The phylogenetic tree was generated using FastTreeMP^51^ using a generalised time-reversible model and then was reconstructed using ClonalFrameML (version 1.11)^57^ to account for recombination. The tree was annotated using iTOL (version 4.3.3)^58^ and Adobe Illustrator (Adobe Inc.).

### Estimate of phylogenetic relationships among streptococci

A phylogenetic tree was constructed using concatenated sequence data from 53 ribosomal loci among all streptococcal genomes in the study data set using the BIGSdb PhyloTree plugin. The tree was graphically simplified to the species level by collapsing clades containing genomes from the same species into a single leaf using iTOL.

### Bacterial strains, media and growth conditions

Pneumococci were cultured in the presence of 5% CO_2_ at 37 °C on Columbia agar (Oxoid) supplemented with 5% horse blood, or in Todd-Hewitt broth supplemented with 0.5% yeast-extract (THY; Oxoid). Mutant strains were selected by using 150 µg/ml spectinomycin. Growth of pneumococci in broth was monitored by measuring optical density at 580 nm (OD_580_) and stocks of pneumococci were stored as single-use 0.5 ml aliquots of THY broth culture (OD_580_ 0.4–0.5) at −70 °C in 10% glycerol. Data for growth curve measurements were collected using 96-well plates in a Tecan Spark microtiter plate reader^59^, measuring the optical density at 595 nm (OD_595_) in 30 min intervals. For growth in THY and serum, 10^6^ CFU of each strain was added to 200 µl of medium or serum and incubated at 37 °C plus 5% CO_2_.

### Construction of Δ*vapE* and Δ*SpnSP38* pneumococcal mutant strains

Strains, plasmids and primers used for this study are described in Supplementary Table 2. Both mutants, ∆*vapE* and *∆SpnSP38*, were generated by overlap extension PCR^60,61^ in the pneumococcal serotype 6B BHN418 strain (a gift from D Ferreira; multilocus sequence type (ST)138) using a transformation fragment in which the *Spn_00749* gene (*vapE*) or the entire satellite prophage, *Spn_00738-Spn_00753*, were replaced by the spectinomycin resistance cassette *aadA9*. For the satellite prophage, two products corresponding to 762 bp upstream (primers SpnSP_UpF and SpnSP_UpspecR) and 872 bp downstream (primers SpnSP_Downspec_F and SpnSP_DownR) of the satellite prophage were amplified from pneumococcal genomic DNA by PCR carrying 3′ and 5′ linkers complementary to the 5′ and 3′ portion of the *aacA9* gene respectively. *aadA9* was amplified from the pR412 plasmid (a gift from M Domenech) using PCR and primers SpnSP_Upspec_F and SpnSP_Downspec_R^60^.

Similarly, for the in-frame deletion of *vapE*, a construct was created in which 820 bp of flanking DNA upstream of the *vapE* ATG (primers VapE_UpF and VapE_UpspcR) and 526 bp of flanking DNA downstream from the *vapE* ORF (starting from the ATG of the overlapping Spn_00750 ORF, primers VapE_DownspecF and VapE_DownR) were amplified by PCR and fused with the *aadA9* cassette by overlap extension PCR^62^. The resulting constructs were then transformed into the BHN418 strain by homologous recombination and allelic replacement using a mix of CSP-1 and CSP-2 and standard protocols^63,64^. The mutations were confirmed by PCR analysis and sequencing.

### Experimental models of infection

Six-week-old female CD-1 mice were obtained from Charles River Laboratory and bred in a conventional animal facility at University College of London (UCL). All animal procedures were conducted in accordance with the United Kingdom (UK) national guidelines for animal use and care and were approved by the UCL Biological Services Ethical Committee and the UK Home Office (Project Licence PPL70/6510). Studies investigating pneumococcal sepsis or pneumonia were performed using 6-week-old mice and infected as previously described^65^.

In brief, in the sepsis model, mice were challenged with 5 × 10^6^ CFU/ml of the serotype 6B strain or the correspondent mutants in a volume of 150 µl by the intraperitoneal route, whereas for pneumonia, mice under anaesthesia with isofluorane were inoculated intranasally with 50 µl containing 10^7^ CFU/mouse of the serotype 6B strain or the mutants. A lethal dose of pentobarbital was administered at 24 or 28 h after challenge and bacterial counts were determined from samples recovered from lung and blood. Lungs and spleens were homogenised through a 0.2 µm filter. Results were expressed as log_10_ CFU/ml of bacteria recovered from the different sites.

For mixed infection experiments, mice were inoculated with a 50/50 mixture of wild-type and mutant pneumococci. The CI was defined as the ratio of the test strain (mutant strain) compared with the control strain (wild-type strain) recovered from mice, divided by the ratio of the test strain to the control strain in the inoculum^66,67^. A CI of <1 indicates that the test strain is attenuated in virulence compared with the control strain, and the lower the CI the more attenuated the strain. Statistical analyses were performed using analysis of variance (ANOVA) for multiple comparisons. GraphPad Prism 7.0 (GraphPad Software, San Diego, CA) was used for statistical analyses.

### C3b binding to pneumococci

Serum samples from five healthy male volunteer controls (median age 40 y) were obtained according to institutional guidelines and stored as single use aliquots at −70 °C to use as a source of complement. Experiments using human cells were approved by the joint University College London/University College Hospitals National Health Service Trust Human Research Ethics Committee, and informed consent was obtained from all participants. C3b deposition was analysed using a flow cytometry assay^68^. In brief, C3b deposition was investigated by incubating 10^7^ CFU of pneumococci with 10 µl of pooled human serum (diluted to 20% in PBS) for 30 min at 37 °C. C3b bound to the different strains was labelled with 50 µl of a 1/500 dilution of fluorescein isothiocyanate-conjugated polyclonal goat anti-human C3b antibody (ICN) after two washes in PBS-Tween 20 (0.01%). The detection of C3b binding was performed using flow cytometry with gating based on the analysis of at least 10,000 bacteria. Experiments were repeated three times and the results were expressed as the proportion of C3b deposition on the surface of the different mutants compared with the C3b deposition on the 6B wild-type strain.

### Neutrophil-killing assay

Frozen aliquots of pneumococci were thawed and washed twice with PBS-Tween 20 (0.01%) by centrifugation for 5 min at 13,000 rpm. In all, 100 µl of the bacterial suspension, diluted to 10^3^ CFU, was added to each well in the presence of 25% baby rabbit complement. After 30 min of incubation at 37˚C, 100 µl of neutrophils (10^5^ cells) previously isolated from human blood using MACSxpress was added to each well and incubated at 37˚C with shaking. Sample aliquots were taken at 15 and 30 min, spotted onto Columbia blood agar plates and incubated at 37 °C plus 5% CO_2_. Bacterial colony counts were performed after overnight incubation.

### Transcriptomic analyses of prophage gene expression

The RNA sequencing data used in this study were originally generated by Blanchette et al.^39^. In brief, samples were collected in three biological replicates from a pneumococcal strain Sp6A-10 isolate (serotype 6A; ST460) growing in Todd-Hewitt broth either planktonically or in polystyrene six-well plates as 2-day-old biofilms. Total RNA from each sample was extracted and sequenced using the Illumina HiSeq4000 sequencing platform. For use in the current study, raw RNA sequencing data was retrieved from the Gene Expression Omnibus (GEO) repository (http://www.ncbi.nlm.nih.gov/geo/; accession number GSE85196). Reads from the control planktonic (THB_PK1, THB_PK2, THB_PK3) and biofilm (THB_BF1, THB_BF2, THB_BF3) samples were paired and mapped onto the pneumococcal Sp6A-10 genome using Bowtie2^69^ with the highest sensitivity option. Differential gene expression and statistical significance (genes with an adjusted *p* value < 0.001 were deemed to be differentially expressed) was computed in Geneious using the DESeq2 method^70^. A volcano plot was generated within the Geneious environment and further edited using Adobe Illustrator.

### Reporting summary

Further information on research design is available in the Nature Research Reporting Summary linked to this article.

## Supplementary information

Supplementary Information

Description of Additional Supplementary Files

Supplementary Data 1

Supplementary Data 2

Supplementary Data 3

Supplementary Data 4

Supplementary Data 5

Supplementary Data 6

Reporting Summary

Source Data

## Data Availability

The 1306 bacterial genomes analysed in this study are available from the rMLST database or PubMLST databases and the corresponding accession numbers are listed in Supplementary Data 2. The 763 full-length and satellite prophage sequences analysed in this study are available in GenBank and the corresponding accession numbers are listed in Supplementary Data 3. The sequence of the *vapE* gene is available via GenBank accession number QBX13222.1.

## Code availability

The PhageMiner and PhageContentCalculator scripts are available without restrictions from GitHub [https://github.com/RezaRezaeiJavan/PhageMiner]; [https://github.com/RezaRezaeiJavan/PhageContentCalculator].
