# Supplementary material for: Prophages and satellite prophages are widespread in Streptococcus and may play a role in pneumococcal pathogenesis
Source: Nat Commun. 2019 Oct 24;10:4852. doi: 10.1038/s41467-019-12825-y (PMC6813308; doi:10.1038/s41467-019-12825-y)
Supplement: Supplementary file 1 — Supplementary Information [file 41467_2019_12825_MOESM1_ESM.pdf]

Supplementary information for:

Prophages and satellite prophages are widespread in *Streptococcus* and may play a role in pneumococcal pathogenesis

Rezaei Javan et al

Supplementary Table 1. Distribution of clusters of orthologous group (COG) categories for all streptococcal genes (i.e. genome-wide) compared to those flanking the prophage.

**Type**	**Functional categories**	**Flanking prophage genes**		***Streptococcus* spp genes***		**Difference**
		**Total (n)**	**Percentage**	**Total (n)**	**Percentage**	
Cellular processes and signalling	[O] Posttranslational modification, protein turnover, chaperones	226	3.5%	3,930	3.2%	
Cellular processes and signalling	[M] Cell wall/membrane/envelope biogenesis	221	3.4%	6,492	5.2%	
Cellular processes and signalling	[V] Defense mechanisms	216	3.3%	4,119	3.3%	
Cellular processes and signalling	[U] Intracellular trafficking, secretion, and vesicular transport	151	2.3%	1,657	1.3%	
Cellular processes and signalling	[T] Signal transduction mechanisms	112	1.7%	3,187	2.6%	
Cellular processes and signalling	[D] Cell cycle control, cell division, chromosome partitioning	68	1.0%	1,367	1.1%	
**Subtotal:**		**994**	**15.3%**	**20,752**	**16.7%**	-1.4%
Information storage and processing	[L] Replication, recombination and repair	818	12.6%	10,255	8.2%	
Information storage and processing	[J] Translation, ribosomal structure and biogenesis	619	9.5%	10,082	8.1%	
Information storage and processing	[K] Transcription	579	8.9%	9,066	7.3%	
**Subtotal:**		**2,016**	**31.0%**	**29,403**	**23.6%**	7.4%
Metabolism	[G] Carbohydrate transport and metabolism	418	6.4%	9,779	7.9%	
Metabolism	[P] Inorganic ion transport and metabolism	403	6.2%	5,868	4.7%	
Metabolism	[E] Amino acid transport and metabolism	385	5.9%	9,704	7.8%	
Metabolism	[F] Nucleotide transport and metabolism	214	3.3%	4,308	3.5%	
Metabolism	[I] Lipid transport and metabolism	130	2.0%	2,472	2.0%	
Metabolism	[C] Energy production and conversion	115	1.8%	4,295	3.5%	
Metabolism	[H] Coenzyme transport and metabolism	97	1.5%	2,804	2.3%	
Metabolism	[Q] Secondary metabolites biosynthesis, transport and catabolism	27	0.4%	744	0.6%	
**Subtotal:**		**1,789**	**27.5%**	**39,974**	**32.1%**	-4.6%
Poorly characterised	[S] Function unknown	1,707	26.2%	34,377	27.6%	-1.4%
**Total****		**6,506**	**100%**	**124,526**	**100%**	

*Sum total of genes in each category, as represented by a dataset consisting of one reference genome for each of 70 different *Streptococcus* spp.

**Chi square test of 2x4 contingency table (flanking prophage genes vs streptococcal genes among four COG groups): p value <0.00001

Supplementary Table 2. Pneumococcal strain, plasmid and primers used in this study.

Name		Description (source/reference/sequence)
**Plasmid**	pR412	Derived from ColE1, carrying a 1145 bp minitransposon that contains Himar1 IRs flanking *addA9* gene. Spc^R (Martin, B., Prudhomme, M., Alloing, G., Granadel, C., Claverys, J.P. 2000)
**Strain**	6B(BHN418)*	*S. pneumoniae* capsular serotype 6
**Primers**	SpnSP_UpF	GGTTTTCATGATGTTGTTCTGG
	SpnSP_Upspec_F	GAGAGAAAAACTTGTTTCATGATCCCCCGTTTGATTTTTAAT
	SpnSP_UpspecR	TTAAAAATCAAACGGGGGATCATGAAACAAGTTTTTCTCT
	SpnSP_Downspec_F	ATTGGATCCATTCCGCGTCTTTAACCTTACCACGGAATTA
	SpnSP_Downspec_R	TAATTCCGTGGTAAGGTTAAAGACGCGGAATGGATCCAAT
	SpnSP_DownR	GATAGCTCCATGTCCGTTGATAC
	SpnSP_Up confirmation	GAAAAGACCATGGTTGGGAT
	SpnSP_Down confirmation	GTTCAGGTAACTCCAAAACC
	VapE_UpF	GTTCCTGAAGGGGCAGATATTG
	VapE_UpspecF	TAAAAAGTGAAATCTTGGAGGTAGATCCCCCGTTTGATTTTT
	VapE_UpspcR	AAAAATCAAACGGGGGATCTACCTCCAAGATTTCACTTTTTA
	VapE_DownspecF	TTGGATCCATTCCGCGTCGTGTGACGTTCTTTTTTT
	VapE_Downspec R	CAAAAAAAGAACGTCACACGACGCGGAATGGATCCAA
	VapE_DownR	CTGTATAATACCAATACGATAGCC
	VapE Up confirmation	GACATCTGGAAGTTTTTGGGG
	VapE Down confirmation	CCTGGTTGTTTGTGGTAGTCT

*This strain was a kind gift from Prof Daniela Ferreira, Liverpool School of Tropical Medicine.

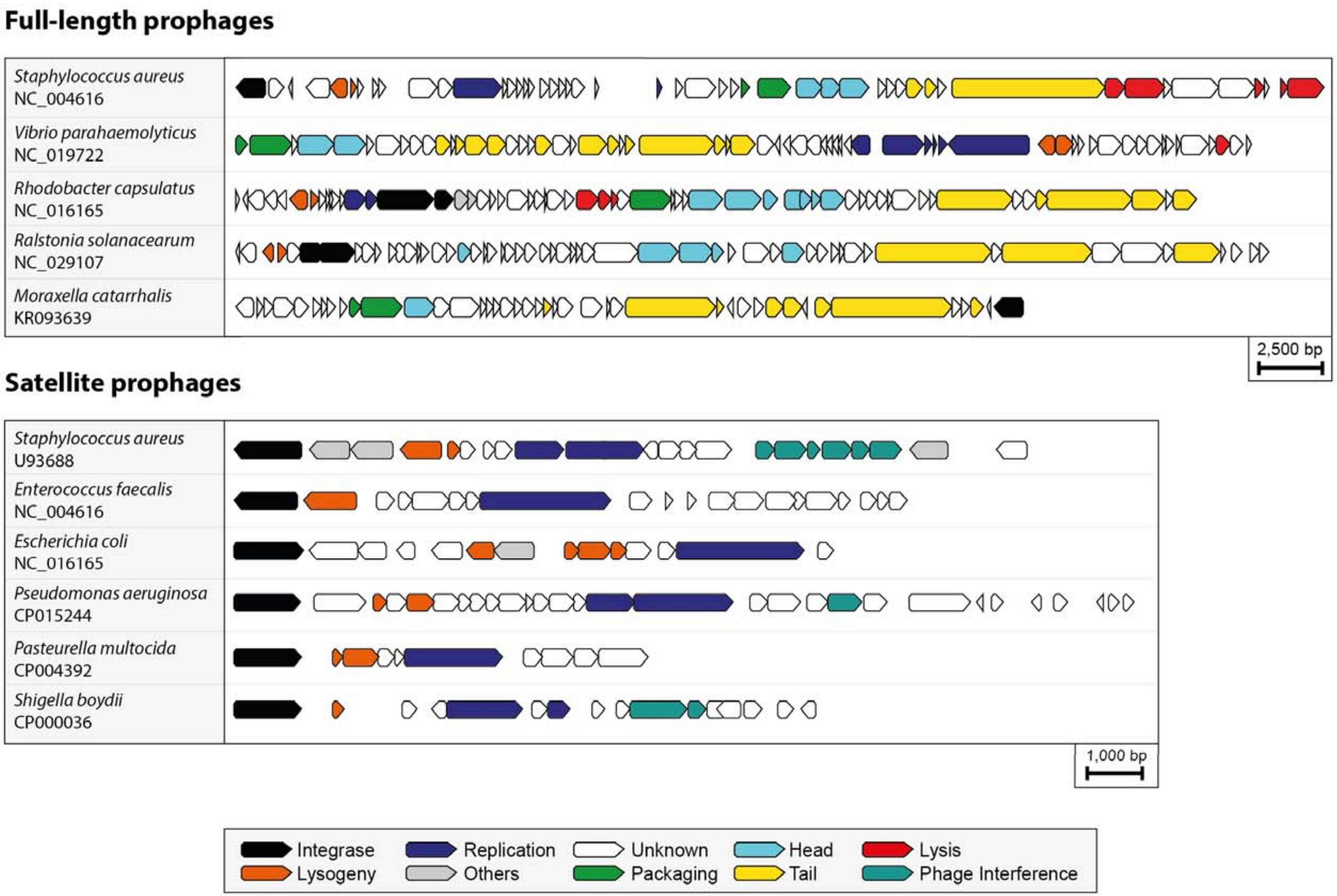

Supplementary Figure 1. Full-length and satellite prophages in different bacterial species. The diagrams illustrate the similarity in gene composition and synteny among different prophages identified in a variety of unrelated bacterial species.

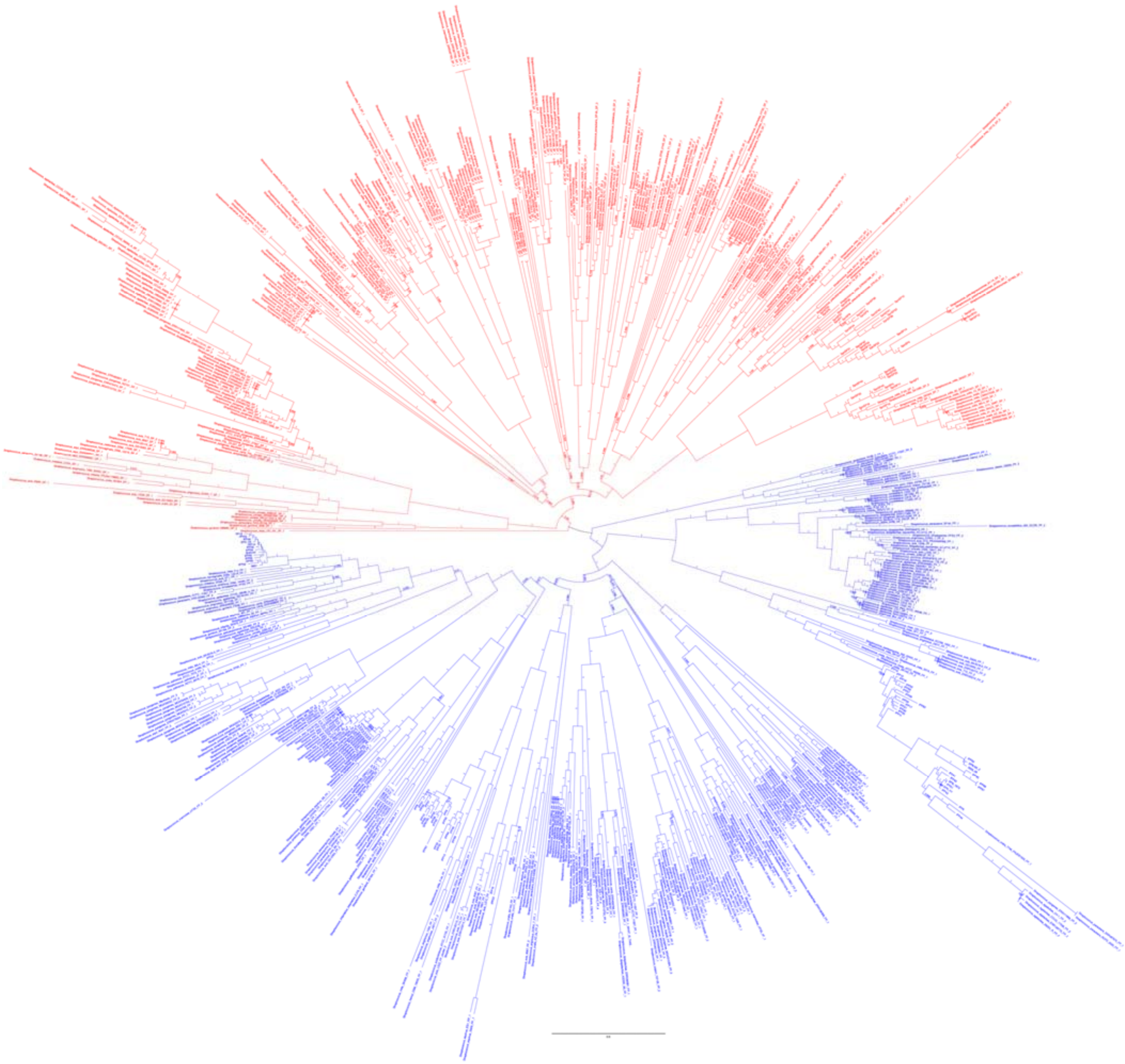

Supplementary Figure 2. Prophages identified among streptococcal genomes. An unrooted phylogenetic tree of all streptococcal prophage genomes identified in the dataset. Blue branches mark full-length prophages and red branches mark satellite prophages. Note that this is the annotated version of Figure 1d in the main paper.

	S_pseudopneumoniae_CCUG_62647_FP_1	S_pseudopneumoniae_5247_FP_1	S_pseudopneumoniae_SRR387783_FP_1	S_pseudopneumoniae_SRR387784_FP_2	S_pseudopneumoniae_IS7493_FP_2	S_infantis_SK970_FP_3	S_oralis_AZ_3a_FP_1	S_oralis_ATCC_49296_FP_2	S_parasanguinis_512_SPAR_FP_cluster_1
S_pseudopneumoniae_CCUG_62647_FP_1		84.7%	100%	100%	71.4%	49.2%	42.9%	42.4%	38.7%
S_pseudopneumoniae_5247_FP_1	84.7%		84.7%	84.7%	51.7%	47.8%	42.3%	41.8%	38.6%
S_pseudopneumoniae_SRR387783_FP_1	100%	84.7%		100%	71.4%	49.2%	42.9%	42.4%	38.7%
S_pseudopneumoniae_SRR387784_FP_2	100%	84.7%	100%		71.4%	49.2%	42.9%	42.4%	38.7%
S_pseudopneumoniae_IS7493_FP_2	71.4%	51.7%	71.4%	71.4%		43.4%	33.2%	35.4%	25.9%
S_infantis_SK970_FP_3	49.2%	47.8%	49.2%	49.2%	43.4%		51.1%	49.1%	40.9%
S_oralis_AZ_3a_FP_1	42.9%	42.3%	42.9%	42.9%	33.2%	51.1%		63.9%	39.5%
S_oralis_ATCC_49296_FP_2	42.4%	41.8%	42.4%	42.4%	35.4%	49.1%	63.9%		37.4%
S_parasanguinis_512_SPAR_FP_1	38.7%	38.6%	38.7%	38.7%	25.9%	40.9%	39.5%	37.4%	
S_parasanguinis_139.rep1_SPAR_FP_1	37.7%	37.6%	37.7%	37.7%	26.5%	39.9%	38.3%	36.9%	74.2%
S_mitis_B9_FP_1	38.0%	38.0%	38.0%	38.0%	26.7%	39.9%	38.7%	37.2%	71.6%
S_parasanguinis_318_SPAR_FP_2	38.1%	38.3%	38.1%	38.1%	26.1%	40.1%	38.4%	36.9%	69.1%
S_pyogenes_ERR448936_FP_2	29.8%	29.8%	29.8%	29.8%	24.3%	29.9%	30.3%	29.6%	31.8%
S_dysgalactiae_ERR109312_FP_1	29.8%	29.8%	29.8%	29.8%	24.3%	29.9%	30.3%	29.6%	31.8%
S_dysgalactiae_ERR085058_FP_1	31.0%	31.1%	31.0%	31.0%	26.2%	31.4%	31.5%	30.3%	33.2%
S_equi_EQUI0022_FP_2	30.0%	30.0%	30.0%	30.0%	24.3%	30.4%	30.8%	29.9%	31.9%
S_suis_YS39_FP_1	28.7%	29.1%	28.7%	28.7%	21.8%	28.1%	28.2%	27.3%	28.8%
S_iniae_SF1_FP_2	28.9%	28.9%	28.9%	28.9%	25.1%	30.0%	29.8%	28.7%	29.0%

	S_parasanguinis_139.rep1_SPAR_FP_1	S_mitis_B9_FP_1	S_parasanguinis_318_SPAR_FP_2	S_pyogenes_ERR448936_FP_2	S_dysgalactiae_ERR109312_FP_1	S_dysgalactiae_ERR085058_FP_1	S_equi_EQUI0022_FP_2	S_suis_YS39_FP_1	S_iniae_SF1_FP_2
S_pseudopneumoniae_CCUG_62647_FP_1	37.7%	38.0%	38.1%	29.8%	29.8%	31.0%	30.0%	28.7%	28.9%
S_pseudopneumoniae_5247_FP_1	37.6%	38.0%	38.3%	29.8%	29.8%	31.1%	30.0%	29.1%	28.9%
S_pseudopneumoniae_SRR387783_FP_1	37.7%	38.0%	38.1%	29.8%	29.8%	31.0%	30.0%	28.7%	28.9%
S_pseudopneumoniae_SRR387784_FP_2	37.7%	38.0%	38.1%	29.8%	29.8%	31.0%	30.0%	28.7%	28.9%
S_pseudopneumoniae_IS7493_FP_2	26.5%	26.7%	26.1%	24.3%	24.3%	26.2%	24.3%	21.8%	25.1%
S_infantis_SK970_FP_3	39.9%	39.9%	40.1%	29.9%	29.9%	31.4%	30.4%	28.1%	30.0%
S_oralis_AZ_3a_FP_1	38.3%	38.7%	38.4%	30.3%	30.3%	31.5%	30.8%	28.2%	29.8%
S_oralis_ATCC_49296_FP_2	36.9%	37.2%	36.9%	29.6%	29.6%	30.3%	29.9%	27.3%	28.7%
S_parasanguinis_512_SPAR_FP_1	74.2%	71.6%	69.1%	31.8%	31.8%	33.2%	31.9%	28.8%	29.0%
S_parasanguinis_139.rep1_SPAR_FP_1		75.9%	66.9%	31.1%	31.1%	32.1%	31.7%	27.9%	28.3%
S_mitis_B9_FP_1	75.9%		67.1%	30.4%	30.4%	31.4%	31.2%	28.0%	28.3%
S_parasanguinis_318_SPAR_FP_2	66.9%	67.1%		30.9%	30.9%	32.3%	31.6%	28.6%	27.9%
S_pyogenes_ERR448936_FP_2	31.1%	30.4%	30.9%		100.0%	84.0%	78.9%	42.9%	36.1%
S_dysgalactiae_ERR109312_FP_1	31.1%	30.4%	30.9%	100.0%		84.0%	78.9%	42.9%	36.1%
S_dysgalactiae_ERR085058_FP_1	32.1%	31.4%	32.3%	84.0%	84.0%		74.2%	44.4%	37.3%
S_equi_EQUI0022_FP_2	31.7%	31.2%	31.6%	78.9%	78.9%	74.2%		44.0%	33.4%
S_suis_YS39_FP_1	27.9%	28.0%	28.6%	42.9%	42.9%	44.4%	44.0%		30.8%
S_iniae_SF1_FP_2	28.3%	28.3%	27.9%	36.1%	36.1%	37.3%	33.4%	30.8%	

Supplementary Figure 3. Genetic similarity among prophages highlighted in Figure 2b. Pairwise comparisons between eighteen full-length prophage sequences were made and the percentage nucleotide similarity is given for each pair of prophages. Cells are shaded grey based on level of similarity, increasing to black for pairs of sequences that are 100% identical.

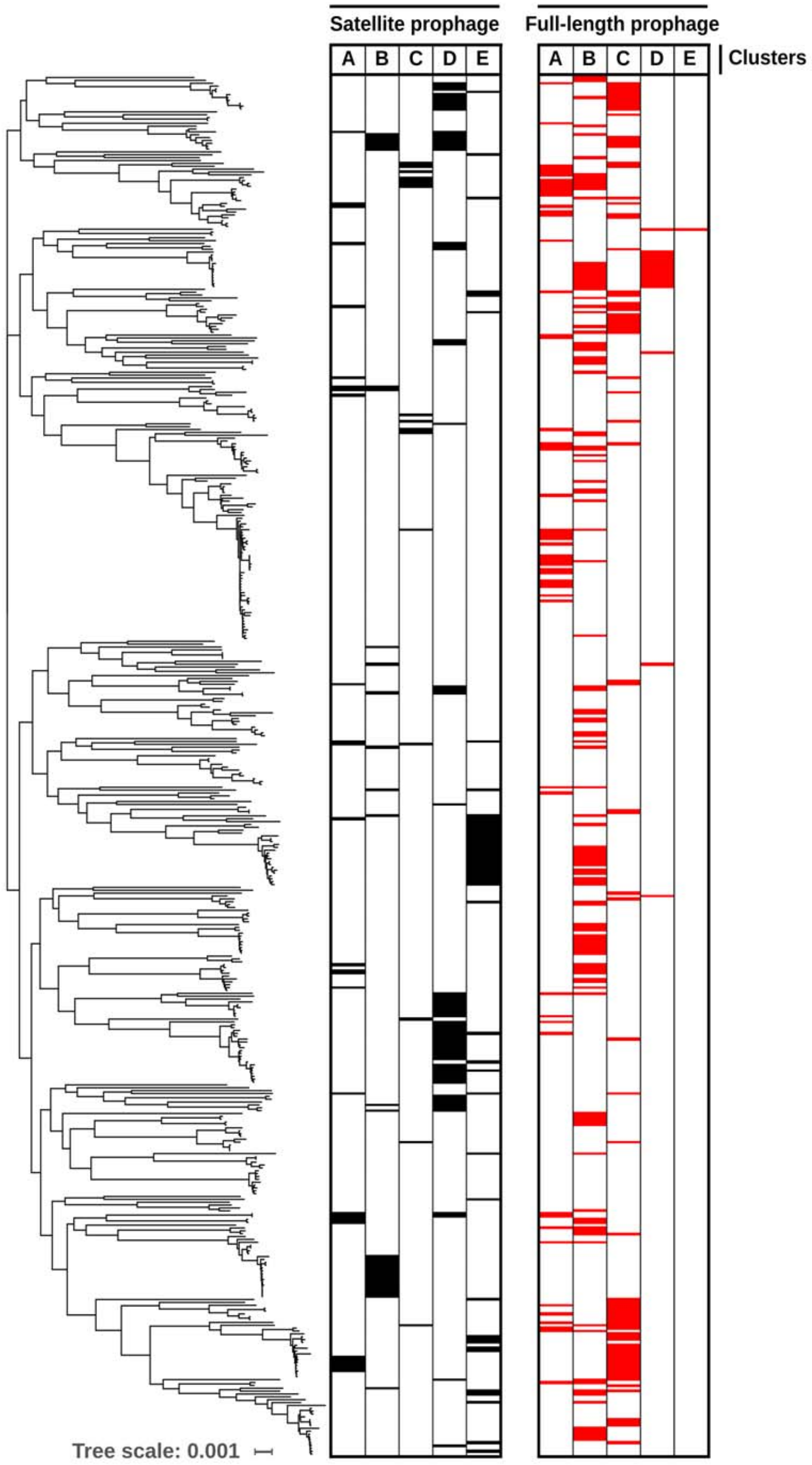

Supplementary Figure 4. A pneumococcal core genome phylogenetic tree. The tree is annotated with the corresponding satellite and full-length prophage clusters.

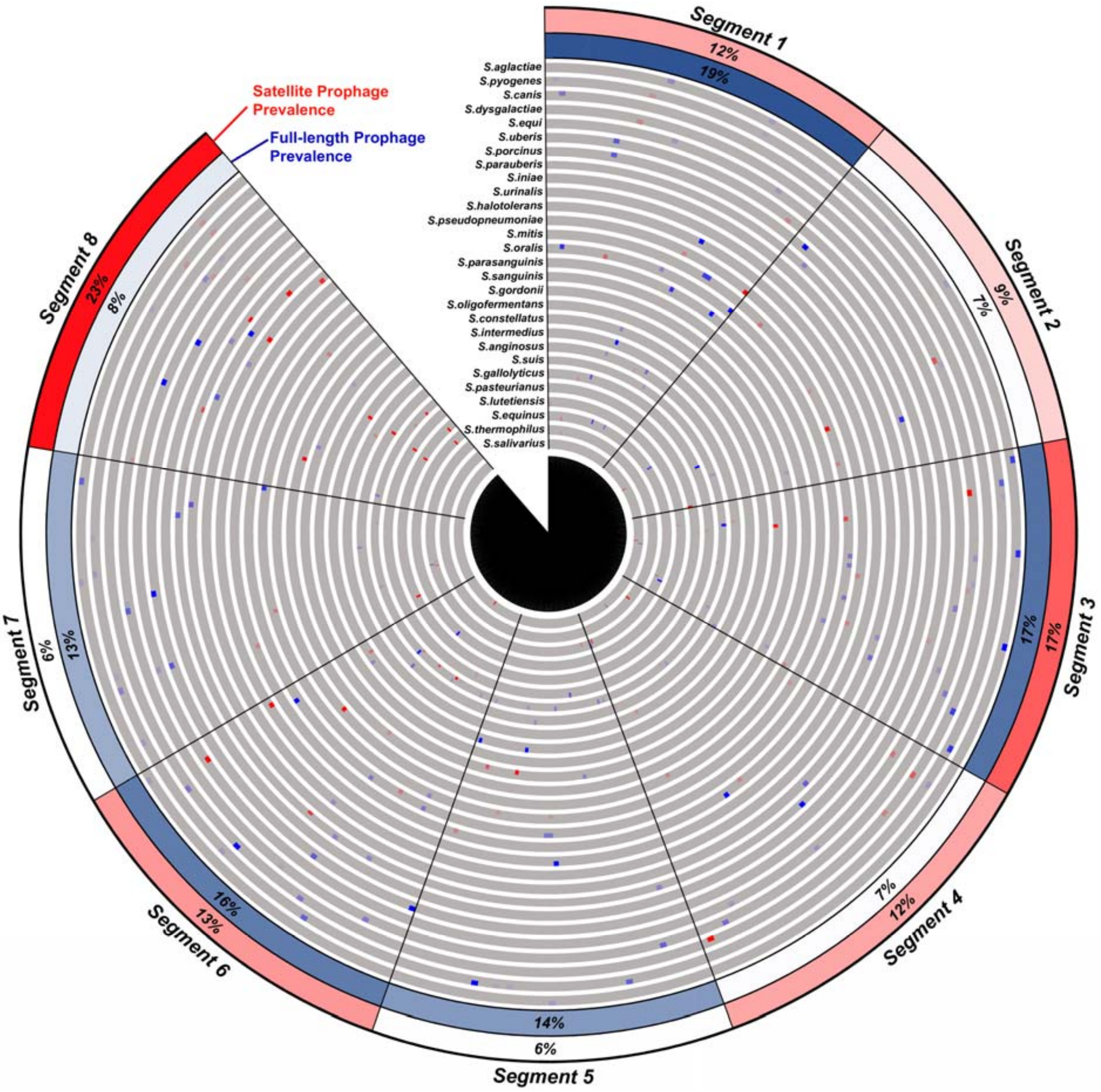

Supplementary Figure 5. Prophage insertion sites within bacterial genomes. One finished genome of each of 29 streptococcal species was divided into eight non-overlapping segments of equal length according to the number of base pairs, and the percentages of prophages situated in each segment were quantified. Source data are provided as a Source Data file.

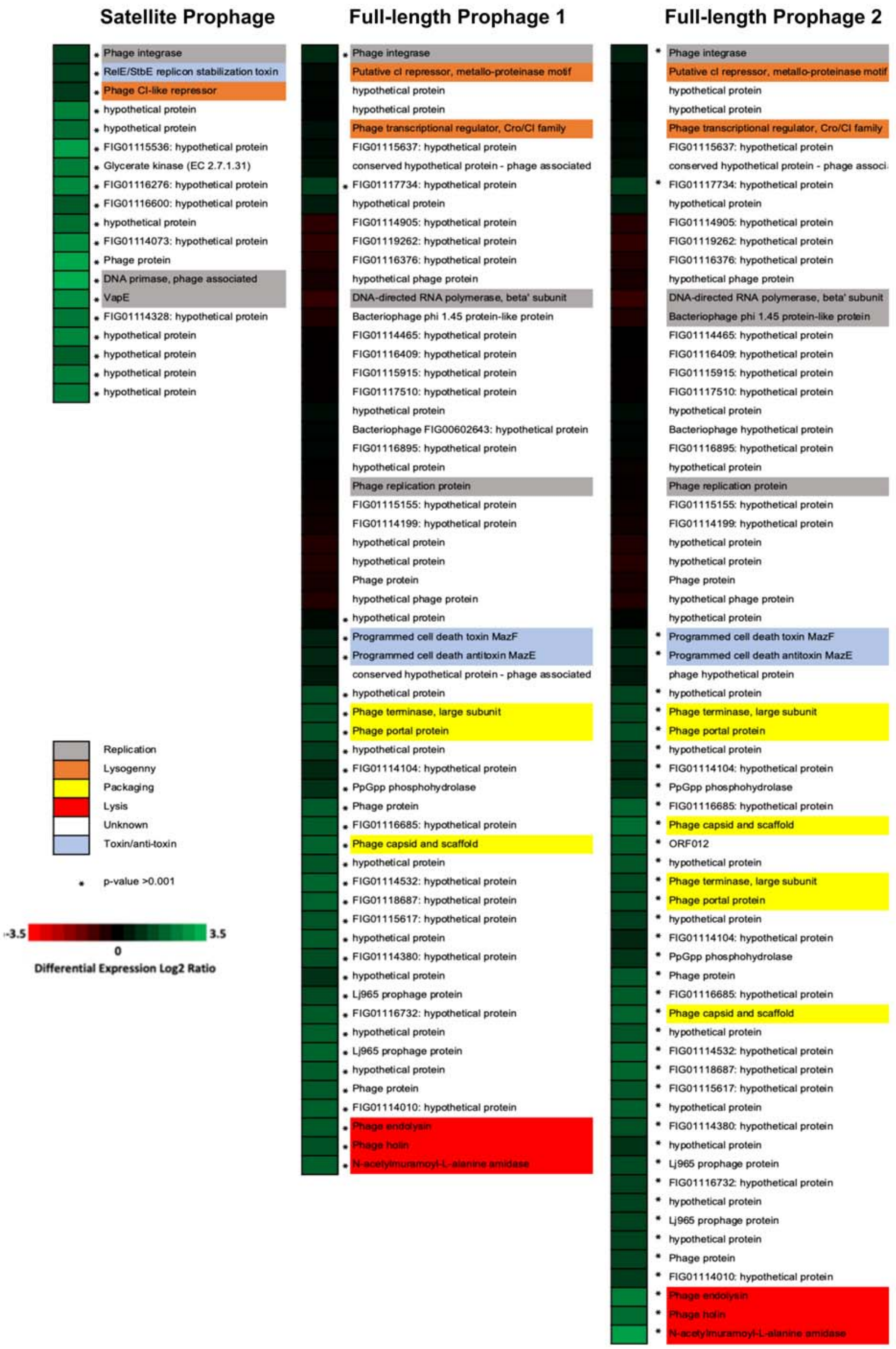

Supplementary Figure 6. Differential expression levels of three prophages. Prophage genes were annotated and the differential expression level of each gene during planktonic growth relative to growth in a biofilm is marked by shades of red (down-regulated genes) or green (up-regulated genes). Differential gene expression and statistical significance was computed in Geneious [https://www.geneious.com] using the DESeq2 method (see Methods). An asterisk to the right of a cell indicates a statistically-significant differential level of expression ($p < 0.05$).
