# Supplementary material for: Prophages and satellite prophages are widespread in Streptococcus and may play a role in pneumococcal pathogenesis
Source: Nat Commun. 2019 Oct 24;10:4852. doi: 10.1038/s41467-019-12825-y (PMC6813308; doi:10.1038/s41467-019-12825-y)
Supplement: Supplementary file 2 — Description of Additional Supplementary Files [file 41467_2019_12825_MOESM2_ESM.pdf]

Description of Additional Supplementary Files

File Name: Supplementary Data 1
Description: **Summary of the prophages found among 70 different streptococcal species.** The table lists the total number of genomes analysed per streptococcal species, with further details about the full-length and satellite prophages harboured within each streptococcal species.

File Name: Supplementary Data 2
Description: **Estimation of the prophage gene content within each streptococcal genome.** Every genome analysed in this study is listed in the spreadsheet and the assembled genome sequence can be accessed from one of three different databases, as indicated. Details of the overall prophage content within each genome are also given.

File Name: Supplementary Data 3
Description: **List of all prophages identified in this study, the insertion site and the host bacterial genes flanking the prophage sequence.** The spreadsheet lists all full-length and satellite prophages identified in this study, including the GenBank accession numbers for each sequence. The five annotated streptococcal genes that flank each end of the prophage sequence are also given.

File Name: Supplementary Data 4
Description: **Genes present in the genome of pneumococcal strain BHN418.** Genes present in the genome of pneumococcal strain BHN418 were annotated and are listed in the order they are found within the genome. The nucleotide and amino acid sequences for each gene are also given.

File Name: Supplementary Data 5
Description: **RNAseq data for all genes in the pneumococcal strain Sp6A-10.** A detailed list of all genes in the Sp6A-10 strain is provided, including the differential expression levels and amino acid sequences.

File Name: Supplementary Data 6
Description: **Descriptive data for the genomes included in the pneumococcal dataset.** Provenance data for each genome are given, along with the serotype, multilocus sequence type allelic profile and clonal complex designation for each genome.
