# Supplementary material for: Prophages and satellite prophages are widespread in Streptococcus and may play a role in pneumococcal pathogenesis
Source: Nat Commun. 2019 Oct 24;10:4852. doi: 10.1038/s41467-019-12825-y (PMC6813308; doi:10.1038/s41467-019-12825-y)
Supplement: Supplementary file 3 — Supplementary Data 1 [file 41467_2019_12825_MOESM3_ESM.pdf]

Supplementary Data 1. Summary of the prophages found among 70 different streptococcal species.

Host species			Full-length prophages				Satellite prophages			
Name	Genomes (n)	Avg. GC content	Unique phages (n)	Avg. size (bp)	Avg. GC content	Avg. no. of genes	Unique phages (n)	Avg. size (bp)	Avg. GC content	Avg. no. of genes
S. pneumoniae	482	39.6%	66	37,346	39.6%	56	44	12,936	37.2%	21
S. pyogenes	50	38.4%	67	39,113	38.5%	56	21	12,825	36.3%	23
S. agalactiae	50	35.3%	37	37,393	39.4%	51	19	14,096	35.7%	23
S. dysgalactiae	50	39.3%	34	39,344	38.6%	57	22	13,455	35.5%	24
S. suis	50	41.2%	28	35,696	41.1%	52	24	10,216	38.1%	17
S. equi	50	41.6%	18	38,096	39.0%	51	4	12,813	36.3%	20
S. parauberis	16	35.5%	14	38,455	35.8%	55	17	13,564	32.7%	20
S. mitis	49	40.1%	12	37,559	40.0%	55	35	12,074	36.2%	18
S. oralis	49	41.0%	12	35,654	39.7%	50	14	11,649	36.0%	17
S. anginosus	24	38.6%	9	37,077	39.6%	51	16	10,921	36.8%	17
S. equinus	27	37.3%	10	40,614	39.1%	56	13	10,075	34.2%	15
S. constellatus	10	38.0%	10	43,497	39.9%	51	4	10,347	35.3%	21
S. uberis	13	36.5%	9	39,936	37.7%	54	11	11,652	33.2%	18
S. gallolyticus	17	37.5%	8	37,207	38.1%	52	9	9,759	35.1%	14
S. canis	11	39.5%	8	37,776	40.4%	51	3	13,895	36.3%	25
S. urinalis	4	34.1%	7	38,072	37.2%	53	7	9,251	34.1%	14
S. parasanguinis	31	41.8%	7	38,470	41.4%	54	6	9,435	34.4%	11
S. gordonii	22	40.4%	7	35,522	40.1%	47	3	10,069	35.4%	15
S. pseudopneumoniae	16	39.8%	5	36,007	39.7%	62	9	11,870	38.2%	19
S. iniae	8	36.6%	5	35,285	36.8%	52	1	12,203	30.0%	18
S. salivarius	32	39.7%	4	40,116	42.2%	42	4	9,577	37.7%	15
S. infantis	4	39.4%	4	35,734	39.3%	49	1	11,158	38.0%	14
S. porcinus	2	36.7%	4	37,741	39.6%	51	0	-	-	-
S. pseudoporcinus	5	37.2%	3	38,992	38.3%	62	2	8,343	33.8%	19
S. entericus	1	44.6%	2	41,400	43.0%	59	2	10,349	42.4%	18
S. himalayensis	1	41.3%	2	39,219	40.8%	49	2	11,811	38.8%	16
S. marmotae	1	40.9%	2	37,995	43.0%	66	2	14,088	40.6%	21
S. infantarius	2	37.6%	2	33,058	38.4%	48	1	9,627	35.7%	14
S. azizii	3	42.7%	2	43,572	41.2%	58	0	-	-	-
S. henryi	2	38.6%	2	40,013	39.8%	58	0	-	-	-
S. ictaluri	2	38.1%	2	24,012	38.4%	32	0	-	-	-
S. intermedius	9	37.6%	1	33,366	38.0%	49	6	13,935	36.4%	23
S. pasteurianus	5	37.3%	1	35,546	38.0%	45	4	10,524	35.0%	15
S. lutetiensis	2	37.6%	1	37,997	39.0%	51	3	10,881	35.1%	15
S. halotolerans	1	39.2%	1	41,477	38.1%	52	2	13,216	36.9%	20
S. hyovaginalis	1	39.9%	1	39,028	37.6%	60	2	12,180	37.0%	22
S. acidominimus	1	42.6%	1	38,191	40.1%	52	1	9,826	39.5%	15
S. cristatus	4	42.6%	1	38,959	39.8%	52	1	13,630	40.4%	17
S. macedonicus	2	37.5%	1	38,767	38.5%	52	1	10,632	33.8%	14
S. phocae	2	39.5%	1	46,987	37.4%	76	1	12,626	36.2%	20
S. cuniculi	1	43.4%	1	28,958	40.4%	44	0	-	-	-
S. orisratti	1	38.5%	1	37,447	41.2%	42	0	-	-	-
S. porci	1	40.8%	1	40,394	35.3%	45	0	-	-	-
S. thoraltensis	1	38.4%	1	40,339	37.5%	57	0	-	-	-
S. thermophilus	32	39.0%	0	-	-	-	16	8,429	37.3%	14
S. sanguinis	37	43.0%	0	-	-	-	6	11,336	38.5%	18
S. vestibularis	6	39.5%	0	-	-	-	3	10,768	36.5%	14
S. castoreus	1	37.8%	0	-	-	-	2	10,268	36.1%	18
S. merionis	1	41.7%	0	-	-	-	1	10,429	39.5%	17
S. oligofermentans	1	42.1%	0	-	-	-	1	10,319	36.2%	16
S. caballi	1	40.4%	0	-	-	-	1	9,970	38.7%	17
S. plurextorum	1	41.1%	0	-	-	-	1	13,633	35.0%	27
Other *strep.* species	111	-	0	-	-	-	0	-	-	-
**Sum**	**1306**	**-**	**415**	**-**	**-**	**-**	**348**	**-**	**-**	**-**
**Average**	**-**	**39.4%**	**-**	**38,003**	**39.2%**	**54**	**-**	**11,767**	**36.2%**	**19**
