# Supplementary material for: Prophages and satellite prophages are widespread in Streptococcus and may play a role in pneumococcal pathogenesis
Source: Nat Commun. 2019 Oct 24;10:4852. doi: 10.1038/s41467-019-12825-y (PMC6813308; doi:10.1038/s41467-019-12825-y)
Supplement: Supplementary file 9 — Reporting Summary [file 41467_2019_12825_MOESM9_ESM.pdf]

nature research

Corresponding author(s): Prof Angela Brueggemann

Last updated by author(s): 18/09/2019

Reporting Summary

Nature Research wishes to improve the reproducibility of the work that we publish. This form provides structure for consistency and transparency in reporting. For further information on Nature Research policies, see Authors & Referees and the Editorial Policy Checklist.

Statistics

For all statistical analyses, confirm that the following items are present in the figure legend, table legend, main text, or Methods section.

n/a	Confirmed	
☐	☒	The exact sample size (*n*) for each experimental group/condition, given as a discrete number and unit of measurement
☐	☒	A statement on whether measurements were taken from distinct samples or whether the same sample was measured repeatedly
☐	☒	The statistical test(s) used AND whether they are one- or two-sided<br>*Only common tests should be described solely by name; describe more complex techniques in the Methods section.*
☒	☐	A description of all covariates tested
☐	☒	A description of any assumptions or corrections, such as tests of normality and adjustment for multiple comparisons
☒	☐	A full description of the statistical parameters including central tendency (e.g. means) or other basic estimates (e.g. regression coefficient) AND variation (e.g. standard deviation) or associated estimates of uncertainty (e.g. confidence intervals)
☒	☐	For null hypothesis testing, the test statistic (e.g. *F*, *t*, *r*) with confidence intervals, effect sizes, degrees of freedom and *P* value noted<br>*Give P values as exact values whenever suitable.*
☒	☐	For Bayesian analysis, information on the choice of priors and Markov chain Monte Carlo settings
☒	☐	For hierarchical and complex designs, identification of the appropriate level for tests and full reporting of outcomes
☒	☐	Estimates of effect sizes (e.g. Cohen's *d*, Pearson's *r*), indicating how they were calculated

Our web collection on statistics for biologists contains articles on many of the points above.

Software and code

Policy information about availability of computer code

Data collection	Genome data that was not initially generated by us was downloaded from GenBank or the European Nucleotide Archive. All genome accession numbers are available in Supplementary Data 3.
Data analysis	The PhageMiner and PhageContentCalculator scripts are available without restrictions from GitHub [https://github.com/RezaRezaeiJavan/PhageMiner]; [https://github.com/RezaRezaeiJavan/PhageContentCalculator].

For manuscripts utilizing custom algorithms or software that are central to the research but not yet described in published literature, software must be made available to editors/reviewers. We strongly encourage code deposition in a community repository (e.g. GitHub). See the Nature Research guidelines for submitting code & software for further information.

Data

Policy information about availability of data

All manuscripts must include a data availability statement. This statement should provide the following information, where applicable:

- Accession codes, unique identifiers, or web links for publicly available datasets
- A list of figures that have associated raw data
- A description of any restrictions on data availability

The 1,306 bacterial genomes analysed in this study are available from the rMLST database [https://pubmlst.org/rmlst/] or PubMLST databases [https://pubmlst.org/databases/] and the corresponding accession numbers are listed in Supplementary Data 2. The 763 full-length and satellite prophage sequences analysed in this study are available in GenBank and the corresponding accession numbers are listed in Supplementary Data 3. The sequence of the vapE gene is available via GenBank accession number QBX13222.1.

Field-specific reporting

Please select the one below that is the best fit for your research. If you are not sure, read the appropriate sections before making your selection.

☒ Life sciences ☐ Behavioural & social sciences ☐ Ecological, evolutionary & environmental sciences

For a reference copy of the document with all sections, see nature.com/documents/nr-reporting-summary-flat.pdf

Life sciences study design

All studies must disclose on these points even when the disclosure is negative.

Sample size	The main outputs are bacterial colony-forming units (CFU) in target organs, analysed using T tests / Mann Whitney U / Kruskal Wallis tests depending on data distribution and the number of groups. Group size calculations using two-tailed T tests and 80% power, looking for a 1 log10 difference and based on previously-published data are as follows: target organ CFU (log10 SD): Group A 3.5, SD 0.5; Group B 2.5, SD 0.5: sample size 5 per group.
Data exclusions	Two mice in the control group of the sepsis experiment were found dead with no possibility of recovering organs for counts.
Replication	Some experiments were replicated and some were not: if a competitive index (CI) and a CFU experiment showed the same effect we would not replicate it as it would be deemed unnecessary, and we are responsible to the licensing authorities to minimise the numbers of mice that are used in experiments.
Randomization	Mouse cages were randomly allocated to experimental group.
Blinding	In this study the investigators were not blinded to the group allocations, this would have required more lab-based investigators than were available.

Reporting for specific materials, systems and methods

We require information from authors about some types of materials, experimental systems and methods used in many studies. Here, indicate whether each material, system or method listed is relevant to your study. If you are not sure if a list item applies to your research, read the appropriate section before selecting a response.

Materials & experimental systems

n/a	Involved in the study	
☒	☐	Antibodies
☒	☐	Eukaryotic cell lines
☒	☐	Palaeontology
☐	☒	Animals and other organisms
☒	☐	Human research participants
☒	☐	Clinical data

Methods

n/a	Involved in the study	
☒	☐	ChIP-seq
☒	☐	Flow cytometry
☒	☐	MRI-based neuroimaging

Animals and other organisms

Policy information about studies involving animals; ARRIVE guidelines recommended for reporting animal research

Laboratory animals	6-week-old female CD-1 mice were obtained from Charles River Laboratory and bred in a conventional animal facility at University College of London.
Wild animals	The study did not involve wild animals.
Field-collected samples	The study did not involve samples collected from the field.
Ethics oversight	Animal procedures were performed according to United Kingdom (UK) national guidelines for animal use and care and approved by the UCL Biological Services Ethical Committee and the UK Home Office (Project Licence PPL70/6510).

Note that full information on the approval of the study protocol must also be provided in the manuscript.
